# Exposure to disulfiram and incidence of parkinsonism

**DOI:** 10.1186/s12995-025-00454-9

**Published:** 2025-03-12

**Authors:** Angelo d’Errico, Elena Strippoli, Samuel M. Goldman, Paul D. Blanc

**Affiliations:** 1 Epidemiology Unit, Local Health Unit ASL To3, Via Martiri XXX Aprile 30, Collegno (Turin), Piedmont Region 10093 Italy; 2 Division of Occupational, Environmental, and Climate Medicine, University of California San Francisco, San Francisco, CA USA

**Keywords:** Parkinsonism, Disulfiram, Carbon disulfide, Epidemiology, Cox regression

## Abstract

**Background:**

Case reports implicate disulfiram treatment in causing parkinsonism, but these observations lack epidemiological confirmation. Aim of the present study was to estimate the risk of incident parkinsonism associated with disulfiram dispensing in a large Italian population.

**Methods:**

In this observational cohort study, administrative data were used, linking records at the individual level from civic registries, population census, mortality registers, hospital admissions, archives of drug prescriptions, and direct ambulatory drug distribution. Participants included all residents in the Piedmont region of Italy aged ≥ 40 years participating in 2011 census, still resident and alive at the beginning of 2013, followed-up from 2013 to 2019. The outcome was incident parkinsonism identified through multiple prescriptions of levodopa or a hospital admission for Parkinson’s disease or atypical parkinsonism. Exposure to disulfiram and to neuroleptics was assessed through regional drug prescription archives. The association between disulfiram and parkinsonism onset was assessed using Cox proportional hazards models, adjusted for gender, age and neuroleptic use.

**Results:**

The study population included 2,498,491 individuals (mean age: 62 years). During follow-up, 19,072 parkinsonism cases were identified, 8 of whom had been prescribed disulfiram. Exposure to disulfiram was associated with a three-fold increased risk of parkinsonism (HR = 3.10, 95% CI = 1.55–6.21) that remained significant when adjusted for neuroleptic use (HR = 2.04, 95% CI = 1.01–4.10). The association was stronger among persons unexposed to neuroleptics and among those with more than four disulfiram prescriptions.

**Conclusions:**

These results support the hypothesis that disulfiram may cause parkinsonism. Clinicians and drug regulatory agencies should consider parkinsonism when assessing the risks and benefits of disulfiram use.

**Supplementary Information:**

The online version contains supplementary material available at 10.1186/s12995-025-00454-9.

## Background

Disulfiram (tetraethylthiuram disulfide) continues to be used in the medical treatment of alcohol use disorders (AUD) [1, 2]. Its aversive mechanism of action is linked to the inhibition of the enzyme acetaldehyde dehydrogenase, leading to an increase in blood acetaldehyde concentration [3]. Disulfiram increases adherence to alcohol abstinence because acetaldehyde is associated with gastrointestinal complaints (nausea and vomiting), cardiovascular abnormalities (tachycardia, dysrhythmia, hypotension) and adverse respiratory effects (dyspnoea and hyperventilation) [4]. The dosages of disulfiram used in the treatment of AUD generally range between 125 and 500 mg per day, with maintenance therapy that extends over a period of months to years [5].

Aside from the intended acute toxicity of ethanol when combined with disulfiram, the metabolism of the parent drug itself can lead to additional toxic effects. Disulfiram is rapidly reduced in the blood to N, N-diethyldithiocarbamate (DDTC) [6, 7], which slowly decomposes spontaneously to carbon disulfide and diethylamine [8, 9]. Indeed, adherence to a disulfiram regimen can be assessed by measuring exhaled carbon disulfide [10]. Carbon disulfide is a well-established neurotoxicant associated with a wide range of adverse outcomes in occupationally-exposed cohorts. For more than 100 years, parkinsonism has been a particularly notable outcome of carbon disulfide exposure in the workplace [11]. Not surprisingly, sporadic case reports and series have suggested that treatment with disulfiram may cause parkinsonian syndromes [12–16]. To date, however, no epidemiologic studies have been conducted investigating this potential relationship.

The aim of this study was to assess the risk of incident parkinsonism associated with disulfiram prescribing in a large population with multi-year longitudinal follow-up, while also taking into account potential confounding by neuroleptic medications that might lead to overlapping symptoms.

## Methods

### Data collection

#### Data and study population

For this analysis, we drew data from the Longitudinal Study of Piedmont, a health monitoring system based on individual record linkage for all residents in the region (more than 4 million people), including civil registries, population census data, mortality registers, hospital admissions, archives of drug prescriptions, and records of direct ambulatory drug distribution [17]. We used data from 2011 to 2019, except for drug prescriptions, which only were available from 2012 to 2019. 

**Fig. 1:**
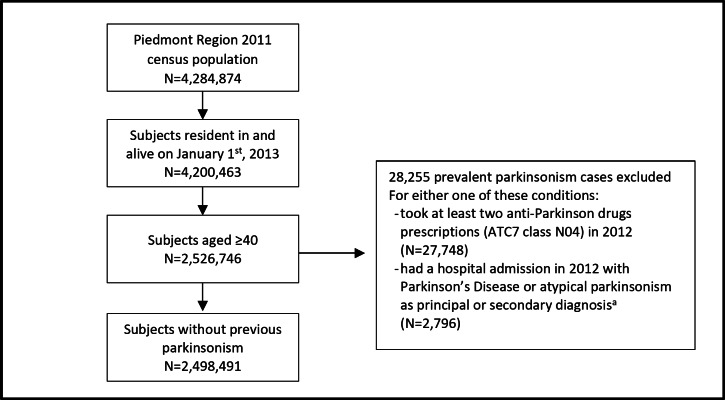
Flowchart of the enrolment of the study population. ^a^ Dementia with Lewy Bodies (ICD-9-CM: 331.82), PD (ICD-9-CM: 332.0x), Corticobasal Degeneration (ICD-9-CM: 331.6x), Multiple System Atrophy and Progressive Supranuclear Palsy (ICD-9-CM:333.0), secondary parkinsonism (ICD-9-CM: 332.1) and essential tremor (ICD-9-CM: 333.1x)

We limited the study cohort to subjects aged ≥ 40 years, interviewed in the 2011 census who were still resident and alive on January 1st, 2013 (*n* = 2,526,746). Prevalent cases at baseline year were excluded (*n* = 28,255) (Fig. 1). Those excluded were individuals who during 2012 received at least two anti-Parkinson drugs prescriptions (any drug in the Anatomical Therapeutic Chemical-ATC class N04), or had a hospital admission with Parkinson’s Disease (PD) or atypical parkinsonism as principal or secondary diagnosis (ICD-9 codes for Dementia with Lewy Bodies (DLB) 331.82, PD 332.0x, Corticobasal Degeneration (CBD) 331.6x, Multiple System Atrophy (MSA) and Progressive Supranuclear Palsy (PSP) 333.0, secondary parkinsonism 332.1 and essential tremor 333.1x).

#### Follow-up and outcome

The study outcome was the occurrence of incident parkinsonism identified through hospital discharge data and drug prescriptions (including ambulatory direct distributions). Using a closed cohort approach, subjects were monitored from January 1st, 2013, until June 30th, 2019, or the date of death or emigration out of the region, whichever occurred first.

**Fig. 2:**
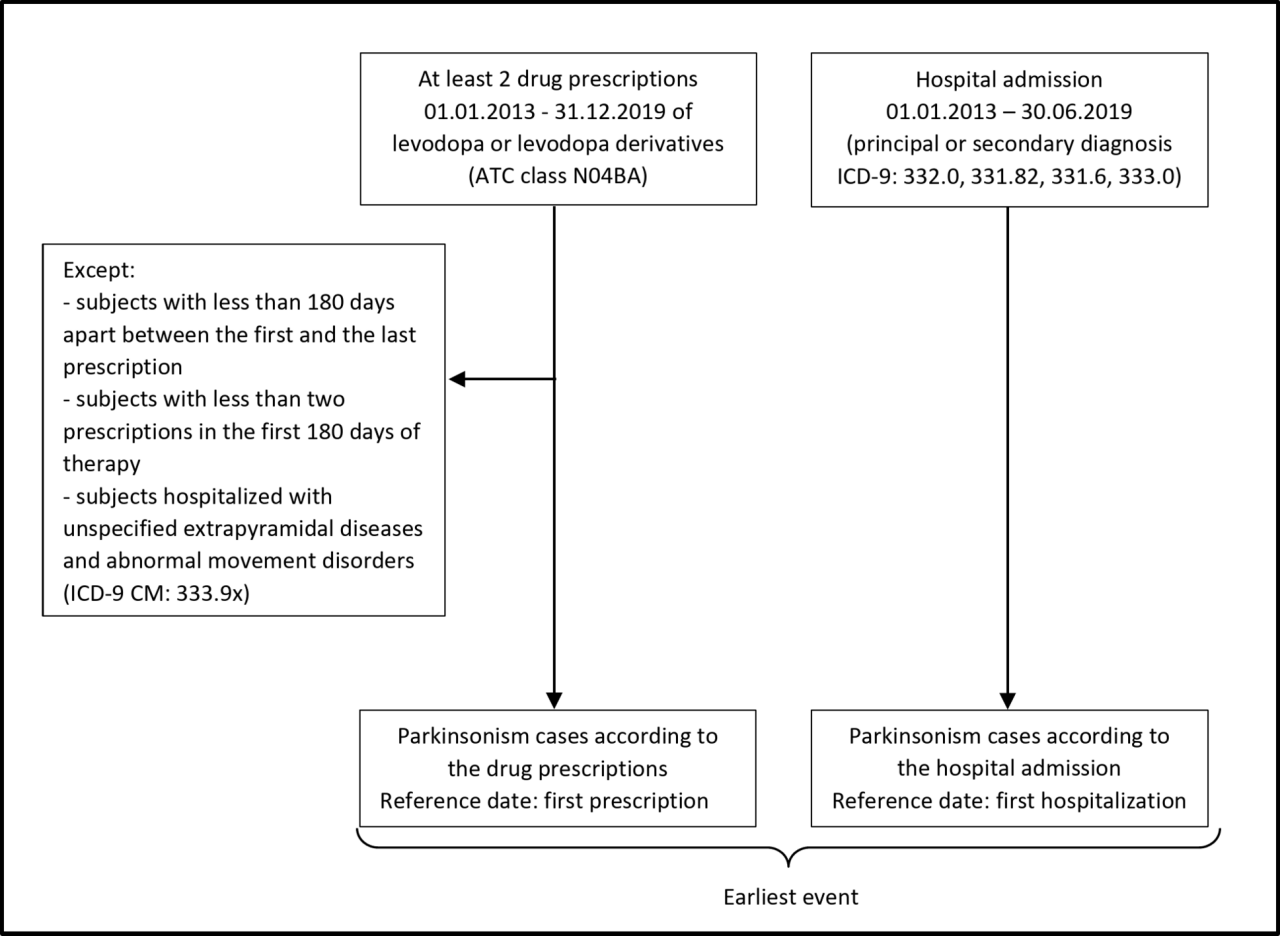
Outline of outcome definition

Incident cases were defined by at least one of the two following criteria: either (i) at least two medication dispensing events of levodopa or levodopa derivatives (ATC class N04BA) during the first 180 days of therapy between 2013 and the end of 2019, and at least 180 days elapsed between the first and the last date of prescription recorded, excluding prescriptions to subjects diagnosed with unspecified extrapyramidal diseases and abnormal movement disorders (ICD-9 CM: 333.9x as principal or secondary diagnosis), or (ii) a hospital admission having as principal or secondary diagnosis ICD-9 codes for PD (332.0), DLB (331.82), CBD (331.6), MSA and PSP (333.0) (Fig. 2). The date of incident illness was the earliest occurrence of hospitalization or first drug prescription.

#### Exposure to disulfiram

Disulfiram exposure was assessed through the Regional ATC Drug Prescription Archives: subjects who received a minimum of two prescriptions of disulfiram (ATC code N07BB01) on different dates were considered exposed, starting from the date of the first prescription. One prescription may include up to two boxes of disulfiram, allowing a duration of the therapy from 15 days to two months. Exposure was ascertained between January 1, 2012 and June 30, 2019, to allow for a minimum of 180 days of levodopa therapy, according to the outcome definition.

#### Exposure to neuroleptics

In light of recognized psychosis comorbidity with alcohol use disorders [18], as well as the risk of parkinsonism associated with previous use of neuroleptics [19, 20], we also carefully considered the use of such drugs in this population. Exposure to neuroleptics was assessed between January 1, 2012 and June 30, 2019, using both the drug prescriptions and the ambulatory distribution archives, defining subjects as exposed if they had at least two prescriptions of any neuroleptic drug on different dates (ATC class N05A).

### Data analysis

A time-span dataset was constructed in order to perform a survival analysis. Person-years contributed to the denominator of non-exposure until the date of first disulfiram prescription (provided that the study participant had at least two prescriptions) and to the denominator of exposure afterwards. Similarly, each individual was considered to be exposed to neuroleptics from the date of the first prescription to the end of follow-up. Age was also treated as a time-varying variable, splitting the observations at each birthday.

The association between disulfiram and parkinsonism onset was assessed using Cox proportional hazards models with robust standard errors. In a first model, the association was estimated adjusting for gender (as strata variable) and age and age squared, to consider eventual non-linear relationship of the HRs of parkinsonism and taking into account model fit with age specified in this manner. In a second model, the analysis was adjusted also for neuroleptic use. In a third model, to assess the possible confounding role of socioeconomic position on the association between disulfiram and parkinsonism, HRs were also adjusted for educational level (high: high school diploma or university degree; intermediate: low-secondary school; low: elementary school or less), used as an indicator of social disadvantage. Additionally, a sensitivity analysis was run stratifying the population by neuroleptic therapy (never exposed vs. ever exposed to such medications). In a further analysis, the risk of parkinsonism was also estimated for exposure to disulfiram by number of prescriptions (2–4, ≥ 5 prescriptions) and the linear trend was tested through ordinal integers representing each prescription category in a Cox regression model. Last, to assess whether results could have been affected by reverse causality, a sensitivity analysis was performed postponing start of exposure to disulfiram by one year. In order to preserve statistical power, these three latter analyses were not adjusted for educational level, after checking in preliminary analyses that education did not influence the association between disulfiram and parkinsonism. No substantial deviation from the proportional hazards assumption was found in the survival analyses for exposure to disulfiram or antipsychotics (Supplementary Fig. 1).

The number-needed-to-harm (NNH) for an event was also computed, using the following formula: $\:NNH=1/(IR\_exposed-IR\_unexposed\:),$ where $\:IR\_exposed\;\mathrm{and}\;IR\_unexposed\:$ are the incidence rates of parkinsonism among exposed and unexposed to disulfiram, standardized by age class (8 classes), exposure to antipsychotics and educational level (3 classes).

Statistical analyses were performed using R version 4.2 (survival package).

## Results

The study cohort was comprised of 2,498,491 persons of whom 53.5% were women; the mean age at the beginning of the study was 62.06 years (sd = 13.56).

The total number of person-years at risk between 2013 and 2019 was 15,025,685, with a mean length of observation of 6.01 years (sd = 1.35). The distribution of basic demographics and exposures are shown in Table 1.

**Table 1:** Descriptive statistics by exposure to disulfiram

	Disulfiram				
	*Exposed*		*Unexposed*		*p*-value
	N	(%)	N	(%)	
**N**	1,178		2,497,313		
**Sex**					< 0.0001
* Male*	818	(69.44)	1,162,148	(46.54)	
* Female*	360	(30.56)	1,335,165	(53.46)	
**Age group (as of January**,** 2013)**					< 0.0001
* 40–44*	192	(16.30)	225,057	(9.01)	
* 45–49*	309	(26.23)	351,237	(14.06)	
* 50–54*	241	(20.46)	313,061	(12.54)	
* 55–59*	182	(15.45)	286,218	(11.46)	
* 60–64*	124	(10.53)	278,768	(11.16)	
* 65–69*	73	(6.20)	260,660	(10.44)	
* 70–74*	39	(3.31)	249,902	(10.01)	
* ≥ 75*	18	(1.53)	532,410	(21.32)	
**Educational attainment**					
* High school diploma or higher*	323	(27.42)	725,819	(29.06)	<0.0001
* Middle school or vocational school diploma*	668	(56.71)	990,510	(39.66)	
* Primary school diploma or less*	187	(15.87)	780,984	(31.27)	
**Neuroleptics** *(exposed)*	350	(29.71)	108,959	(4.36)	<0.0001
**Parkinsonism cases**	8	(0.68)	19,064	(0.76)	0.740
*Case definition N (% of cases)*					
* Only hospital admission*,* by diagnosis*:					
* PD*	1	(12.50)	1,336	(7.01)	
* DLB*	0	(0.00)	224	(1.17)	
* MSA and PSP*	0	(0.00)	205	(1.08)	
* CBD*	0	(0.00)	0	(0.00)	
*Only outpatient medication prescription*	5	(62.50)	14,491	(76.01)	
* Drugs and hospital admission*,* by diagnosis*:					
* PD*	2	(25.00)	2,463	(12.92)	
* DLB*	0	(0.00)	103	(0.54)	
* MSA and PSP*	0	(0.00)	242	(1.27)	
* CBD*	0	(0.00)	0	(0.00)	
*Parkinsonism cases ever exposed to neuroleptics*	2	(25.00)	2,423	(12.71)	

* PD* Parkinson’s Disease

*MSA* Multiple System Atrophy

*BLB* Dementia with Lewy Bodies

*PSP* Progressive Supranuclear Palsy

*CBD* Corticobasal Degeneration

There were 1,178 (0.05%) persons in the cohort who had been exposed to disulfiram. Exposure to disulfiram was more frequent among men (0.07%) than women (0.027%), as well as among subjects of younger age (almost 78% of exposed subjects but only 47% of unexposed subjects were less than age 60 at baseline).

During follow-up, 19,072 (0.76%) cases of parkinsonism were identified (Table 1), with an incidence rate of 12.69 cases per 10,000 person-years (95% CI = 12.51–12.87). Most of the cases were not hospitalized and were identified only through levodopa therapy (*N* = 14,491, 75.98%); 1,771 (9.29%) had at least one hospitalization associated with parkinsonism without levodopa therapy; and 2,810 (14.73%) experienced both hospitalization associated with parkinsonism and levodopa drug prescription. During follow-up, the median duration of levodopa therapy was 2.41 years (IQR = 1.27–3.92). Eight subjects who were exposed to disulfiram developed incident parkinsonism, with a median time interval between the first disulfiram prescription and the occurrence of the outcome of 931 days (IQR = 333–1404). None of the eight exposed cases had been exposed to other drugs for alcohol use disorders, such as acamprosate, naltrexone and others (ATC code: N07BB), during the observation period.

Of these eight, seven received levodopa therapy for a median of 2.82 years during the observation period; one was hospitalized with Parkinson’s disease as the primary diagnosis. Among the parkinsonism cases, 2,425 (12.71%) patients had ever taken neuroleptics prior to disease onset.

Table 2 provides the results of the survival analysis. In analyses adjusted for sex and age (Model 1), exposure to disulfiram was associated with a three-fold increased risk of parkinsonism (HR = 3.10, 95% CI = 1.55–6.21). Disulfiram and neuroleptic therapy were strongly associated with each other (350 in the disulfiram group [29.71%] were also prescribed a neuroleptic; *p* < 0.001). Consistent with this, two of the eight parkinsonism cases among the disulfiram users had also been prescribed a neuroleptic medication. When neuroleptic drug prescription was included in the regression model, the point estimate of disulfiram risk decreased from three-fold to two-fold, but remained statistically significant (HR = 2.04, 95% CI = 1.01–4.09) (Model 2). In that model (Model 2), neuroleptic use was also independently associated with a higher risk of parkinsonism (HR = 4.09, 95% CI: 3.94–4.36). The further adjustment for educational level did not modify the association between disulfiram and parkinsonism (Model 3). Postponing the start of exposure 1-year after the first prescription slightly attenuated the association (HR = 1.751, 95% CI = 0.784–3.910), though it was no longer statistically significant due to the lower number of exposed cases (Table 3).

**Table 2:** Hazard ratios (HR) of parkinsonism by exposure to disulfiram and neuroleptics therapy

		*N* parkinsonism cases	Person-years	Model 1^a^		Model 2^b^		Model 3^c^	
				HR	95% CI	HR	95% CI	HR	95% CI
**Disulfiram**	*Unexposed*	19,064	15,020,303	1		1		1	
	*Exposed*	8	5,382	3.103	1.551–6.207	2.037	1.013–4.095	2.040	1.015–4.101
**Neuroleptics**	*Unexposed*	16,665	14,670,915			1		1	
	*Exposed*	2,407	354,770			4.095	3.984–4.362	4.180	3.995–4.374

^a^ adjusted for sex (strata), age and age squared

^b^ adjusted for sex (strata), age, age squared and neuroleptic exposure

^c^ adjusted for sex (strata), age, age squared, neuroleptic exposure and educational attainment

**Table 3:** Hazard ratios (HR) of parkinsonism by exposure to disulfiram postponed 1-year after the first prescription and neuroleptics therapy

		N parkinsonism cases	Person-years	Model 1^a^		Model 2^b^	
				*HR*	*95% CI*	*HR*	*95% CI*
**Disulfiram** (lagged 1 year from the first prescription)	*Unexposed*	19,066	15,021,186	1		1	
	*Exposed*	6	4,499	2.702	1.215–6.010	1.751	0.784–3.910
**Neuroleptics**	*Unexposed*	16,665	14,670,915			1	
	*Exposed*	2,407	354,770			4.170	3.986–4.364

^a^ adjusted for sex (strata), age and age squared

^b^ adjusted for sex (strata), age, age squared and neuroleptic exposure

In an analysis stratified by neuroleptic use (Table 4), disulfiram-associated risk for parkinsonism was elevated in the non-neuroleptic-exposed (HR = 3.42, 95% CI: 1.54–7.62), whereas there was no increased risk associated with disulfiram in the population co-exposed to neuroleptics (two observed cases only; HR = 0.64, 95% CI: 0.16–2.59).

**Table 4:** Hazard ratios (HR) of parkinsonism by exposure to disulfiram therapy, stratified by exposure to neuroleptics

		Ever exposed to Neuroleptics				Never exposed to Neuroleptics			
		*N* parkinsonism cases	Person-years	HR^a^	95% CI	*N* parkinsonism cases	Person-years	HR^a^	95% CI
**Disulfiram**	*Unexposed*	2,423	573,126	1		16,641	14,447,177	1	
	*Exposed*	2	1,693	0.644	0.160–2.586	6	3,981	3.422	1.537–7.621
**Total**		2,425	574,819			16,647	14,450,866		

^a^ adjusted for sex (strata), age and age squared

Examining the relationship between incident parkinsonism and exposure to disulfiram by number of prescriptions, we observed a higher risk estimate among those with more than four prescriptions (*p*-value for trend 0.02) (Table 5). Further, this exposure-response remained after restricting the analysis to subjects not exposed to neuroleptics (HR = 5.79 95%CI = 2.19–15.32, *p*-value for trend < 0.01) (data not shown).

**Table 5:** Hazard ratios (HR) of parkinsonism by number of prescriptions of disulfiram

	*N* parkinsonism cases	Person-years	Model 1^a^		Model 2^b^	
			HR	95% CI	HR	95% CI
Number of disulfiram prescriptions						
*Unexposed*	19,064	15,020,303	1		1	
*2–4 prescriptions*	4	3,285.21	2.486	0.928–6.658	1.553	0.579–4.166
*> 4 prescriptions*	4	2,096.60	4.128	1.561–10.916	2.958	1.109–7.893
*Trend test (p-value)*			< 0.001		0.022	

^a^ adjusted for sex (strata), age and age squared

^b^ adjusted for sex (strata), age, age squared and antipsychotic exposure

The computation of the number-needed-to-harm (NNH) for an event showed that it would be necessary to treat 849 patients with disulfiram to produce one additional case of parkinsonism.

## Discussion

In this study, we observed a two- to three-fold increased risk of parkinsonism associated with disulfiram, increasing to up to four-fold with a greater number of disulfiram prescriptions. Moreover, the excess risk we observed did not appear to be accounted for by concomitant neuroleptic exposure and socioeconomic position. The absence of prescriptions of other drugs used to treat alcohol disorders among cases exposed to disulfiram during the observation period indicates that also these drugs are unlikely confounders of the observed association. The exclusion of a confounding effect by these drugs is relevant, in particular for acamprosate, for which several case reports have documented the development of parkinson-like or extrapyramidal syndromes [21–23].

These findings support previous case-based observations suggesting that treatment with disulfiram may be causally related with parkinsonism [14–16]. The observed association of neuroleptics with parkinsonism also is consistent with previous data [20] and supports the population-based analytic approach that we applied to this study question.

Parkinsonism includes a group of syndromes characterized by impaired motion, tremor, muscle rigidity and postural instability. These syndromes can be classified as primary or secondary based on aetiology. Among primary parkinsonism, Parkinson’s Disease (PD) is the most frequent form, followed by Multiple System Atrophy (MSA), Dementia with Lewy Bodies (DLB), Progressive Supranuclear Palsy (PSP) and Corticobasal Degeneration (CBD) [24, 25]. Primary parkinsonism syndromes are caused by neurodegenerative processes linked to the intracellular deposition of the amyloidogenic proteins alpha-synuclein (for PD, DLB and MSA) and tau (for PSP and CBD) in brain structures [26, 27]. For the most part, secondary parkinsonian syndromes are manifestations of medication side effects of dopamine receptor-blocking neuroleptics [28]. Drug-induced parkinsonism is the most frequent cause of parkinsonism after PD [29] and may not be easily distinguishable from PD or other primary forms of parkinsonism [30, 31]. Thus, drug-induced parkinsonism may be misclassified as PD or atypical parkinsonism [20]. For this reason, our analysis employed conservative strategies to account for possible confounding by neuroleptic prescribing.

Various acute and subacute neurological syndromes, including dysarthria, ataxia, encephalopathy, tremor, seizures, paresis, and peripheral neuropathies have been reported due to intoxication with disulfiram [32–37]. Support for a causal effect of disulfiram in the development of these syndromes derives from the fact that in most cases they improved or disappeared after withdrawal of the drug and that in different studies pathological alterations in the basal ganglia or in the mid-brain were identified at MRI or CT scan in patients with CNS involvement [14–16, 35], comparable to those found in patients affected by PD or atypical parkinsonism [38]. Although the mechanism of action of disulfiram has not been fully elucidated, its neurotoxicity has been attributed to its metabolite N, N-diethyldithiocarbamate (DDTC), which has been shown to produce neurological effects in humans and in different animal species on both the peripheral and the central nervous system. These include peripheral axonopathy characterized by neurofilament alterations and demyelinization [39–42], and neurological deficits at clinical examination as well as myelopathy, with axonal degeneration in the posterior columns of the spinal cord [39, 43] and alterations in the cytoskeleton proteins of glial neurons [44, 45]. However, DDTC has also been shown to inhibit the enzyme dopamine-beta-hydroxylase, which catalyzes the transformation of dopamine into norepinephrine in the brain, increasing dopamine brain concentration through an accumulation mechanism [46, 47]. This finding appears in contradiction with the reduced dopaminergic transmission typical of parkinsonism and other extrapyramidal syndromes induced by drugs [20], suggesting that DDTC is unlikely the metabolite directly responsible for these syndromes, and that other disulfiram metabolites are involved.

In particular, the parkinsonism-inducing effects of DDTC may be mediated by its breakdown to carbon disulfide based on the observation that it produces neurological effects similar to those of DDTC. Further, blood concentrations of carbon disulfide increase substantially after ingestion of disulfiram [8, 48–50]. For example, in one study, blood concentration of carbon disulfide increased by more than 40-fold after oral administration of 250–500 mg/day disulfiram [48]. Carbon disulfide is a solvent which has been employed since the mid-nineteenth century in cold vulcanization of rubber; at the turn of the twentieth century, it was introduced into the production of rayon and other cellulose-based viscose processes. Occupational exposure to carbon disulfide is known to cause parkinsonism as well as psychosis and peripheral neuropathies [51, 52]. The mechanisms through which carbon disulfide acts as a neurotoxicant leading to parkinsonism include damage to dopaminergic neurons in the substantia nigra [14, 15, 53, 54].

### Strengths

This population-based observational study of parkinsonism risk associated with disulfiram therapy addresses a critical gap in the epidemiology of this important adverse drug effect. The large cohort we analyzed includes all residents in the Piedmont Region of Italy followed-up for multiple years and allows the investigation of this relationship in a manner that accounts for the low incidence of parkinsonism in the general population and the very small proportion of persons treated with disulfiram.

Among other strengths, the study population included the whole resident population in the region, which minimizes the likelihood of selection bias, and plausibly allows generalization of the results to the Italian general population of corresponding age. Also, the availability of objective information on disulfiram therapy, parkinsonism incidence, and the use of neuroleptics, based on administrative data, reduces the likelihood of differential misclassification of the exposures of interest or of the outcome having biased risk estimates.

### Limitations

One key potential limitation in this study is that the association observed between disulfiram and parkinsonism could have been attributable to alcohol use disorder itself rather than to disulfiram prescribing given that disulfiram is a drug specifically used to treat alcoholism. Thus, it is not possible to disentangle the independent effect of alcoholism and disulfiram on the risk of parkinsonism. Alcoholism has been reported to damage neurons in the basal ganglia and other subcortical structures in animals [55], but no direct relationship between alcohol misuse and parkinsonism has been established. In fact, alcohol consumption has been inversely associated with Parkinson disease in a series of meta-analyses [56–59]. Furthermore, a large U.S. cohort study did not find any association between alcohol consumption greater than 30 g/day and risk of Parkinson’s disease [60]. Regarding the relationship of parkinsonism with alcoholism or heavy alcohol intake, the few available studies also show contradictory findings [61–64].

Another limitation is that we did not have information on the dosage of disulfiram in our data, so that we could not ascertain if the association with parkinsonism was present only for use of elevated doses or also for low dosage. Last, caution is needed in interpreting the results on the association between disulfiram and parkinsonism, although statistically significant, due to the small number of exposed cases observed.

## Conclusions

Despite carbon disulfide’s well-recognized potency as a neurotoxicant and even though carbon disulfide is a major metabolite of disulfiram, this medication still remains in today’s pharmacopeia. This may be due in part to the lack of previous epidemiological studies that have formally assessed the association between disulfiram and parkinsonism. Moreover, the risk may not be receding. Even though disulfiram use for alcohol use disorder has decreased in recent decades, at the same time, this medication has been introduced in cocaine dependence, based on its ability to inhibit the enzyme dopamine β-hydroxylase [46]. Disulfiram also has been considered for use as an antimicrobial agent [3] as well as in cancer chemotherapy [65, 66]. Although the NNH for an event resulted to be relatively high, clinicians and drug regulatory agencies should consider parkinsonism when assessing the risks and benefits of disulfiram use, whether for ethanol use disorder or other indications.

## Supplementary Information

Supplementary Material 1.

Supplementary Material 2.

## Data Availability

The data are not publicly available due to legal restrictions established by the European privacy law, as the data contain potentially identifying or sensitive patient information. Open access to data is not possible, but collaborations in specific projects with other research groups or institutes are possible, upon institutional agreement.
