# Supplementary material for: Exposure to disulfiram and incidence of parkinsonism
Source: J Occup Med Toxicol. 2025 Mar 12;20:8. doi: 10.1186/s12995-025-00454-9 (PMC11899656; doi:10.1186/s12995-025-00454-9)
Supplement: Supplementary file 1 — Supplementary Material 1. [file 12995_2025_454_MOESM1_ESM.docx]

**Online-Only Supplementary Material**

**Exposure to Disulfiram and incidence of Parkinsonism**

**Supplementary Figure 1.** Kaplan-Meier observed survival curves (dashed ones) and Cox predicted curves (solid ones) for disulfiram and antipsychotics exposure.

**Supplementary Figure 1.** Kaplan-Meier observed survival curves (dashed ones) and Cox predicted curves (solid ones) for disulfiram and antipsychotics exposure.^a^


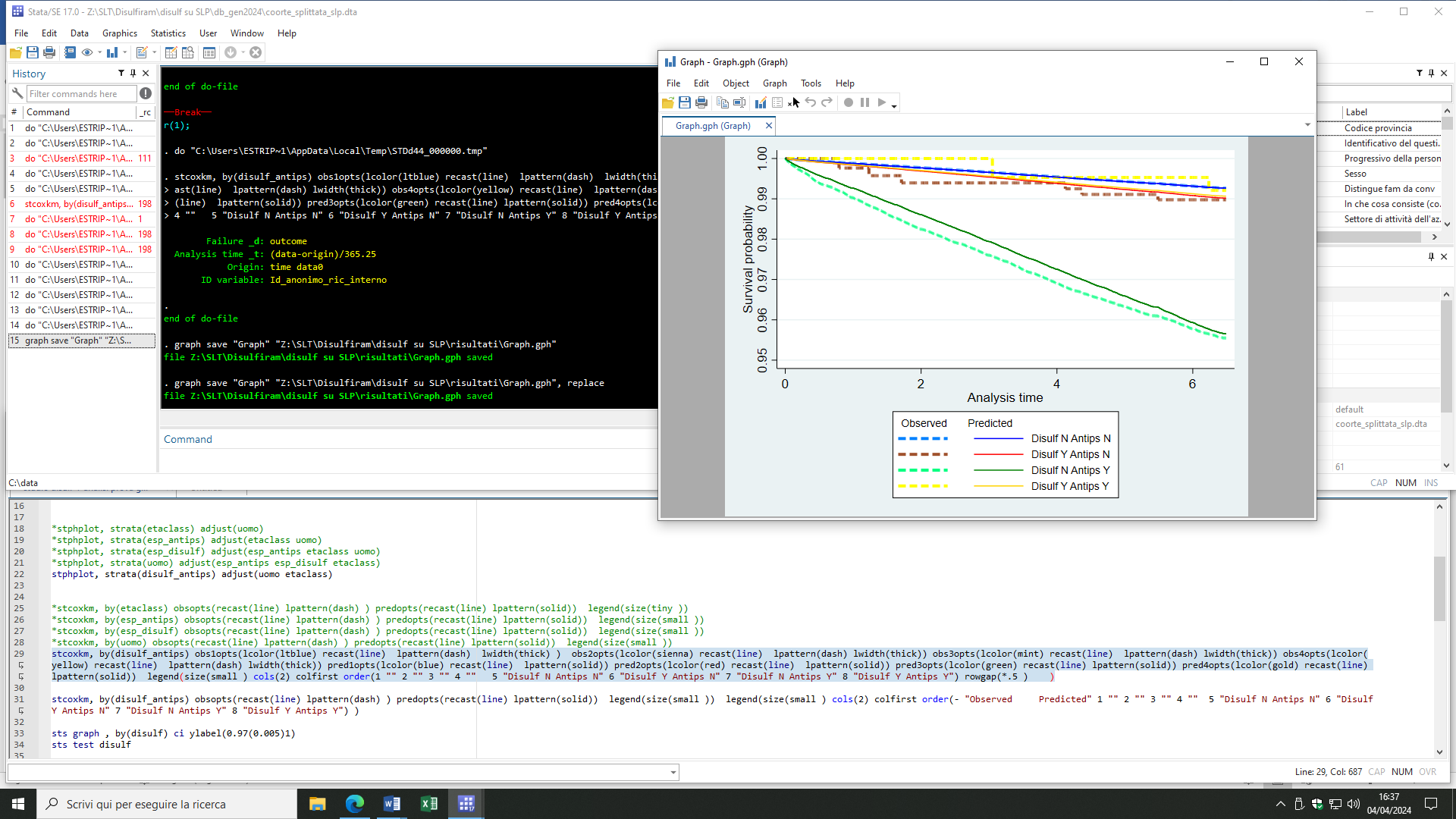


^a^ The closer the observed values are to the predicted, the less likely that the proportional-hazards assumption has been violated. Here the graph shows a not perfect overlapping between the observed and predicted curves among subjects exposed to antispychotics, while perfect overlapping in the unexposed population.
