# Supplementary material for: Exposure to disulfiram and incidence of parkinsonism
Source: J Occup Med Toxicol. 2025 Mar 12;20:8. doi: 10.1186/s12995-025-00454-9 (PMC11899656; doi:10.1186/s12995-025-00454-9)
Supplement: Supplementary file 2 — Supplementary Material 2. [file 12995_2025_454_MOESM2_ESM.docx]

**Exposure to Disulfiram and incidence of Parkinsonism**

**Supplementary Table 1**

STROBE Statement—checklist of items that should be included in reports of observational studies

|  | | | Item No. | Recommendation | Page  No. | | Relevant text from manuscript |
| --- | --- | --- | --- | --- | --- | --- | --- |
| **Title and abstract** | | | 1 | (*a*) Indicate the study’s design with a commonly used term in the title or the abstract | 1 | | “Incidence of parkinsonism”, which implies a cohort study |
|  |  |  |  | (*b*) Provide in the abstract an informative and balanced summary of what was done and what was found | 2 | | “Exposure to disulfiram was associated with a three-fold increased risk of parkinsonism (HR= 3.10, 95% CI=1.55-6.21) that remained significant when adjusted for neuroleptic use (HR=2.04, 95% CI=1.01-4.10).” |
| Introduction | | | | | | |  |
| Background/rationale | | | 2 | Explain the scientific background and rationale for the investigation being reported | 3 | | “Disulfiram is rapidly reduced in the blood to N,N-diethyldithiocarbamate (DDTC) [6,7], which slowly decomposes spontaneously to carbon disulfide and diethylamine [8,9].”  “Carbon disulfide is a well-established neurotoxicant associated with a wide range of adverse outcomes in occupationally-exposed cohorts. Not surprisingly, sporadic case reports and series have suggested that treatment with disulfiram may cause parkinsonian syndromes [12-16].” |
| Objectives | | | 3 | State specific objectives, including any prespecified hypotheses | 3 | | “The aim of this study was to assess the risk of incident parkinsonism associated with disulfiram prescribing in a large population with multi-year longitudinal follow-up.” |
| Methods | | | | | | |  |
| Study design | | | 4 | Present key elements of study design early in the paper | 4 | | “For this analysis, we drew data from the Longitudinal Study of Piedmont, a health monitoring system based on individual record linkage for all residents in the region (more than 4 million people), including civil registries, population census data, mortality registers, hospital admissions, archives of drug prescriptions, and records of direct ambulatory drug distribution [17].” |
| Setting | | | 5 | Describe the setting, locations, and relevant dates, including periods of recruitment, exposure, follow-up, and data collection | 4 | | “We used data from 2011 to 2019, except for drug prescriptions, which only were available from 2012 to 2019.” |
| Participants | | | 6 | (*a*) *Cohort study*—Give the eligibility criteria, and the sources and methods of selection of participants. Describe methods of follow-up  *Case-control study*—Give the eligibility criteria, and the sources and methods of case ascertainment and control selection. Give the rationale for the choice of cases and controls  *Cross-sectional study*—Give the eligibility criteria, and the sources and methods of selection of participants | 4 | | “We limited the study cohort to subjects aged ≥40 years, interviewed in the 2011 census who were still resident and alive on January 1st, 2013 (n= 2,526,746).”  “Prevalent cases at baseline year were excluded (n=28,255). Those excluded were individuals who during 2012 received at least two anti-Parkinson drugs prescriptions (any drug in the Anatomical Therapeutic Chemical-ATC class N04), or had a hospital admission with Parkinson’s Disease (PD) or atypical parkinsonism as principal or secondary diagnosis (ICD-9 codes for Dementia with Lewy Bodies (DLB) 331.82, PD 332.0x, Corticobasal Degeneration (CBD) 331.6x, Multiple System Atrophy (MSA) and Progressive Supranuclear Palsy (PSP) 333.0, secondary parkinsonism 332.1 and essential tremor 333.1x).” |
|  |  |  |  | (*b*) *Cohort study*—For matched studies, give matching criteria and number of exposed and unexposed  *Case-control study*—For matched studies, give matching criteria and the number of controls per case |  | | NA |
| Variables | | | 7 | Clearly define all outcomes, exposures, predictors, potential confounders, and effect modifiers. Give diagnostic criteria, if applicable | 4-5 | | “Incident cases were defined by at least one of the two following criteria: either i) at least two medication dispensing events of levodopa or levodopa derivatives (ATC class N04BA) during the first 180 days of therapy between 2013 and the end of 2019, and at least 180 days elapsed between the first and the last date of prescription recorded, excluding prescriptions to subjects diagnosed with unspecified extrapyramidal diseases and abnormal movement disorders (ICD-9 CM: 333.9x as principal or secondary diagnosis), or ii) a hospital admission having as principal or secondary diagnosis ICD-9 codes for PD (332.0), DLB (331.82), CBD (331.6), MSA and PSP (333.0) (Figure 1). The date of incident illness was the earliest occurrence of hospitalization or first drug prescription.” |
| Data sources/ measurement | | | 8* | For each variable of interest, give sources of data and details of methods of assessment (measurement). Describe comparability of assessment methods if there is more than one group | 6 | | “Disulfiram exposure was assessed through the Regional ATC Drug Prescription Archives: subjects who received a minimum of two prescriptions of disulfiram (ATC code N07BB01) on different dates were considered exposed, starting from the date of the first prescription. Exposure was ascertained between January 1, 2012 and June 30, 2019, to allow for a minimum of 180 days of levodopa therapy, according to the outcome definition.” |
| Bias | | | 9 | Describe any efforts to address potential sources of bias | 6  7 | | “In light of recognized psychosis comorbidity with alcohol use disorders [18], as well as the risk of parkinsonism associated with previous use of neuroleptics [19,20], we also carefully considered the use of such drugs in this population. Exposure to neuroleptics was assessed between January 1, 2012 and June 30, 2019, using both the drug prescriptions and the ambulatory distribution archives, defining subjects as exposed if they had at least two prescriptions of any neuroleptic drug on different dates (ATC class N05A).”  “Last, to assess whether results could have been affected by reverse causality, a sensitivity analysis was performed postponing start of exposure to disulfiram by one year.” |
| Study size | | | 10 | Explain how the study size was arrived at | 5  Figure 1 | | “We limited the study cohort to subjects aged ≥40 years, interviewed in the 2011 census who were still resident and alive on January 1st, 2013 (n= 2,526,746). Prevalent cases at baseline year were excluded (n=28,255) (Figure 1).” |
| Quantitative variables | | 11 | | Explain how quantitative variables were handled in the analyses. If applicable, describe which groupings were chosen and why | 6 | | “A time-span dataset was constructed in order to perform a survival analysis. Person-years contributed to the denominator of non-exposure until the date of first disulfiram prescription (provided that the study participant had at least two prescriptions) and to the denominator of exposure afterwards. Similarly, each individual was considered to be exposed to neuroleptics from the date of the first prescription to the end of follow-up. Age was also treated as a time-varying variable, splitting the observations at each birthday.” |
| Statistical methods | | 12 | | (*a*) Describe all statistical methods, including those used to control for confounding | 6-7 | | “The association between disulfiram and parkinsonism onset was assessed using Cox proportional hazards models with robust standard errors. In a first model, the association was estimated adjusting for gender (as strata variable) and age and age squared, to consider eventual non-linear relationship of the HRs of parkinsonism and taking into account model fit with age specified in this manner. In a second model, the analysis was adjusted also for neuroleptic use. In a third model, to assess the possible confounding role of socioeconomic position on the association between disulfiram and parkinsonism, HRs were also adjusted for educational level (high: high school diploma or university degree; intermediate: low-secondary school; low: elementary school or less), used as an indicator of social disadvantage.” |
|  |  |  |  | (*b*) Describe any methods used to examine subgroups and interactions | 7 | | “Additionally, a sensitivity analysis was run stratifying the population by neuroleptic therapy (never exposed vs. ever exposed to such medications). In a further analysis, the risk of parkinsonism was also estimated for exposure to disulfiram by number of prescriptions (2-4, ≥5 prescriptions) and the linear trend was tested through ordinal integers representing each prescription category in a Cox regression model.” |
|  |  |  |  | (*c*) Explain how missing data were addressed | NA | |  |
|  |  |  |  | (*d*) *Cohort study*—If applicable, explain how loss to follow-up was addressed  *Case-control study*—If applicable, explain how matching of cases and controls was addressed  *Cross-sectional study*—If applicable, describe analytical methods taking account of sampling strategy | NA | |  |
|  |  |  |  | (*e*) Describe any sensitivity analyses | 7 | | “Additionally, a sensitivity analysis was run stratifying the population by neuroleptic therapy (never exposed vs. ever exposed to such medications).” |
| Results | | | | | | | |
| Participants | | 13* | | (a) Report numbers of individuals at each stage of study—eg numbers potentially eligible, examined for eligibility, confirmed eligible, included in the study, completing follow-up, and analysed | 7 | | “The study cohort was comprised of 2,498,491 persons of whom 53.5% were women; the mean age at the beginning of the study was 62.06 years (sd=13.56).” |
|  |  |  |  | (b) Give reasons for non-participation at each stage | NA | |  |
|  |  |  |  | (c) Consider use of a flow diagram | Figure 1 | |  |
| Descriptive data | | 14* | | (a) Give characteristics of study participants (eg demographic, clinical, social) and information on exposures and potential confounders | 8  Table 1 | | “The distribution of basic demographics and exposures are shown in Table 1.” |
|  |  |  |  | (b) Indicate number of participants with missing data for each variable of interest | NA | |  |
|  |  |  |  | (c) *Cohort study*—Summarise follow-up time (eg, average and total amount) | 8 | | “The total number of person-years at risk between 2013 and 2019 was 15,025,685, with a mean length of observation of 6.01 years (sd=1.35).” |
| Outcome data | | 15* | | *Cohort study*—Report numbers of outcome events or summary measures over time | 9 | | “During follow-up, 19,072 (0.76%) cases of parkinsonism were identified (Table 1), with an incidence rate of 12.69 cases per 10,000 person-years (95% CI=12.51-12.87). Most of the cases were not hospitalized and were identified only through levodopa therapy (N=14,491, 75.98%); 1,771 (9.29%) had at least one hospitalization associated with parkinsonism without levodopa therapy; and 2,810 (14.73%) experienced both hospitalization associated with parkinsonism and levodopa drug prescription.” |
|  |  |  |  | *Case-control study—*Report numbers in each exposure category, or summary measures of exposure |  | |  |
|  |  |  |  | *Cross-sectional study—*Report numbers of outcome events or summary measures |  | |  |
| Main results | | 16 | | (*a*) Give unadjusted estimates and, if applicable, confounder-adjusted estimates and their precision (eg, 95% confidence interval). Make clear which confounders were adjusted for and why they were included | 10 | | “In analyses adjusted for sex and age (Model 1), exposure to disulfiram was associated with a three-fold increased risk of parkinsonism (HR= 3.10, 95% CI=1.55-6.21). When neuroleptic drug prescription was included in the regression model, the point estimate of disulfiram risk decreased from three-fold to two-fold, but remained statistically significant (HR=2.04, 95% CI=1.01-4.09). The further adjustment for educational level did not modify the association between disulfiram and parkinsonism (Model 3).” |
|  |  |  |  | (*b*) Report category boundaries when continuous variables were categorized | NA | |  |
| Other analyses | 17 | | Report other analyses done—eg analyses of subgroups and interactions, and sensitivity analyses | | 10-11 | “In an analysis stratified by neuroleptic use (Table 3), disulfiram-associated risk for parkinsonism was elevated in the non-neuroleptic exposed (HR=3.42, 95% CI: 1.54-7.62), whereas there was no increased risk associated with disulfiram in the population co-exposed to neuroleptics (two observed cases only; HR=0.64, 95% CI: 0.16-2.59). Examining the relationship between incident parkinsonism and exposure to disulfiram by number of prescriptions, we observed a higher risk estimate among those with more than four prescriptions (p-value for trend 0.02) (Table 4). Further, this exposure-response remained after restricting the analysis to subjects not exposed to neuroleptics (HR=5.79 95%CI=2.19-15.32, p-value for trend <0.01) (data not shown).” | |
| Discussion | | | | | | | |
| Key results | 18 | | Summarise key results with reference to study objectives | | 11-12 | “In this study, we observed a two- to three-fold increased risk of parkinsonism associated with disulfiram, increasing to up to four-fold with a greater number of disulfiram prescriptions. Moreover, the excess risk we observed did not appear to be accounted for by concomitant neuroleptic exposure and socioeconomic position. The absence of prescriptions of other drugs used to treat alcohol disorders among cases exposed to disulfiram during the observation period indicates that also these drugs are unlikely confounders of the observed association. The exclusion of a confounding effect by these drugs is relevant, in particular for acamprosate, for which several case reports have documented the development of parkinson-like or extrapyramidal syndromes [21-23].” | |
| Limitations | 19 | | Discuss limitations of the study, taking into account sources of potential bias or imprecision. Discuss both direction and magnitude of any potential bias | | 14 | “One key potential limitation in this study is that the association observed between disulfiram and parkinsonism could have been attributable to alcohol use disorder itself rather than to disulfiram prescribing given that disulfiram is a drug specifically used to treat alcoholism.” | |
| Interpretation | 20 | | Give a cautious overall interpretation of results considering objectives, limitations, multiplicity of analyses, results from similar studies, and other relevant evidence | | 12 | “These findings support previous case-based observations suggesting that treatment with disulfiram may be causally related with parkinsonism [14-16]. The observed association of neuroleptics with parkinsonism also is consistent with previous data [20] and supports the population-based analytic approach that we applied to this study question.” | |
| Generalisability | 21 | | Discuss the generalisability (external validity) of the study results | | 14 | “Among other strengths, the study population included the whole resident population in the region aged 40 years or older, which minimizes the likelihood of selection bias and plausibly allows generalization of the results to the Italian general population of corresponding age.” | |
| Other information | | |  | | | | |
| Funding | 22 | | Give the source of funding and the role of the funders for the present study and, if applicable, for the original study on which the present article is based | | NA |  | |
